# Drip fertilization improve water and nitrogen use efficiency by optimizing root and shoot traits of winter wheat

**DOI:** 10.3389/fpls.2023.1201966

**Published:** 2023-06-30

**Authors:** Shoutian Ma, Ye Meng, Qisheng Han, Shouchen Ma

**Affiliations:** ^1^ School of Surveying and Land Information Engineering, Henan Polytechnic University, Jiaozuo, China; ^2^ Institute of Farmland Irrigation, Chinese Academy of Agricultural Sciences (CAAS), Key Lab for Crop Water Requirement and Regulation of Ministry of Agriculture, Xinxiang, China; ^3^ School of Faculty Engineering, University of Putra Malaysia, Selonga, Malaysia

**Keywords:** winter wheat, drip fertilization, root and shoot traits, water utilization efficiency, fertilizer utilization efficiency

## Abstract

Proper irrigation and fertilization measures can not only improve water and fertilizer utilization efficiency, but also have important significance in ensuring agricultural environment security and sustainable development. A field experiment was conducted to determine the optimal drip fertilization measure of winter wheat and explain its mechanism by analyzing the physiological and ecological characteristics and utilization efficiency of water and nitrogen under different irrigation and fertilization methods. The plants were treated with three irrigation and fertilization methods: the traditional irrigation and fertilization method (CK), surface drip fertilization (I1) and underground drip fertilization (I2). The results demonstrated that different irrigation methods had various effects on population and physiological characteristics of wheat. The plant height, leaf area and tiller number of I1 were significantly higher than those of CK during the whole growth period. I2 decreased plant height, leaf area and tiller number at jointing stage, but at flowering stage, the leaf area of I2 t was significantly higher than that of CK. Different irrigation methods also affected the root distribution of wheat. At flowering stage, I1 had lower root biomass than CK in all soil layers. The upper root system of I2 was smaller, but the deep root system was larger compared with the control. I1 and I2 had lower total root weight and higher shoot biomass compared to CK, so their root-shoot ratio decreased significantly. I1 and I2 increased and instantaneous water use efficiency (IWUE) by increasing the photosynthetic rate (Pn) and reducing transpiration rate (Tr) at the flowering stage, while I2 had a similar Pn to I1, but reduced Tr, resulting in a higher IWUE than I1. Both I1 and I2 also increased root efficiency, root activity, and Fv/Fm of wheat at the late growth stage, promoting accumulated dry matter after flowering (ADM) and pre-flowering dry matter remobilization (DMR), leading to a significant increase in grain yield. In addition, I1 and I2 had significantly higher water productivity (WP), irrigation water productivity (IWP), nitrogen partial productivity (NPP) and nitrogen agronomic efficiency (NAE) than CK, especially I2 had the highest IWP, WP, NPP and NAE. These findings highlight the potential benefits of drip fertilization in promoting sustainable wheat production and elucidate the mechanism by which it promotes efficient use of water and fertilizer.

## 1 Introduction

The Huang-Huai-hai Plain is the main wheat producing area in China. As the precipitation in this region is difficult to meet the water demand of winter wheat during the whole growth period, the wheat production mainly relies on groundwater for supplementary irrigation, and the irrigation method is mostly flood irrigation, which results in serious waste of irrigation water (Xu et al., 2019). Moreover, farmers overuse chemical fertilizer to increase crop yields in this region, which not only causes waste of fertilizer resources, but also seriously pollutes the agricultural environment. Proper irrigation and fertilization measures can reduce water and fertilizer losses and thus improve the use efficiency of water and fertilizer (Gheysari et al., 2009). Drip fertilization technology can accurately and timely apply water and fertilizer near root zone of plant through piping system, thus improving grain yield and WP of crop (Wang et al., 2016; Zhang et al., 2017; Cao et al., 2022) and reducing fertilizer loss and improving fertilizer utilization efficiency (Sharmasarkar et al., 2001). At present, researches on drip irrigation mainly focus on cash crops such as cotton (Wang et al., 2020), fruits and vegetables (Al-Ghobari and Dewidar, 2018), while there are few researches on winter wheat and many problems need to be further understood. Therefore, the research on drip fertilization for winter wheat can not only improve water and fertilizer utilization efficiency of crop, but also have important significance to ensure agricultural environment security and sustainable development in the region.

Root system, as the direct absorption part of soil water and nutrients, is also the main consumption organ of photosynthate. Root absorption function affects the development of plant canopy and the utilization efficiency of water and fertilizer resources. Drip fertilization optimizes the distribution characteristics of soil water and fertilizer, which also affect physiological and ecological characteristics of root such as root distribution (Wang et al., 2020) and root activity (Ma et al., 2022), and ultimately affect shoot growth. In addition, in order to maintain root physiological function, the root system consumes about 50% of the photosynthates through root respiration (Liu and Li, 2005). It is clear that reducing carbon consumption in root respiration helps to increase grain yield by through appropriate agricultural measures. Root respiration is greatly affected by water and fertilizer characteristics of soil. Therefore, it is helpful to understand the mechanism of drip fertilization promoting efficient utilization of water and fertilizer by exploring the effects of drip fertilization on root physiological characteristics, such as root respiration and root activity, as well as the response of shoot to root physiological characteristics. However, current researches on drip irrigation mainly concentrated on crop morphological characteristics, water-saving effects, soil nutrient transport, and irrigation system selection (Sun and Ma, 2016). There is a lack of in-depth and systematic research on the response and feedback of plant roots to drip fertilization. Therefore, it is of great significance to systematically study the physiological response of root system to drip fertilization and its regulation on the shoot for understanding the physiological mechanism of drip fertilization promoting efficiency production of crop.

Therefore, in this study, a field experiment was conducted to systematically explored the mechanism of drip fertilization promoting crop efficient production by comparing the physiological and ecological characteristics of the root and shoot (such as root and shoot biomass, root activity, root respiration, photosynthesis, ADM and DMR) under different irrigation and fertilization conditions, in order to provide scientific theoretical basis for the optimization of water-saving irrigation technology for winter wheat.

## 2 Materials and methods

### 2.1 Experimental design and material

**Figure 1:**
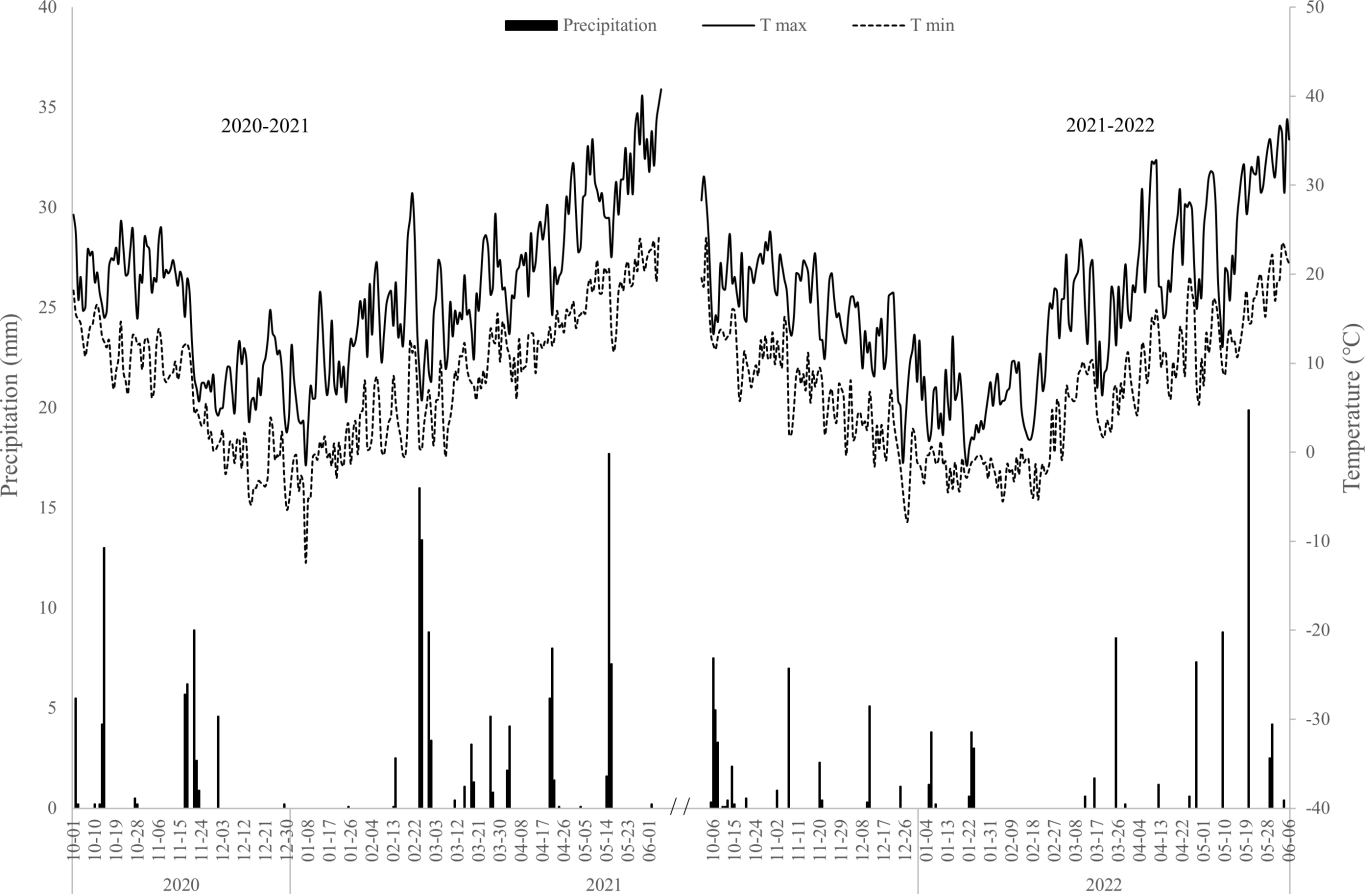
The meteorological information during the winter wheat seasons. Tmax, minimum temperature; Tmin, maximum temperature.

**Figure 2:**
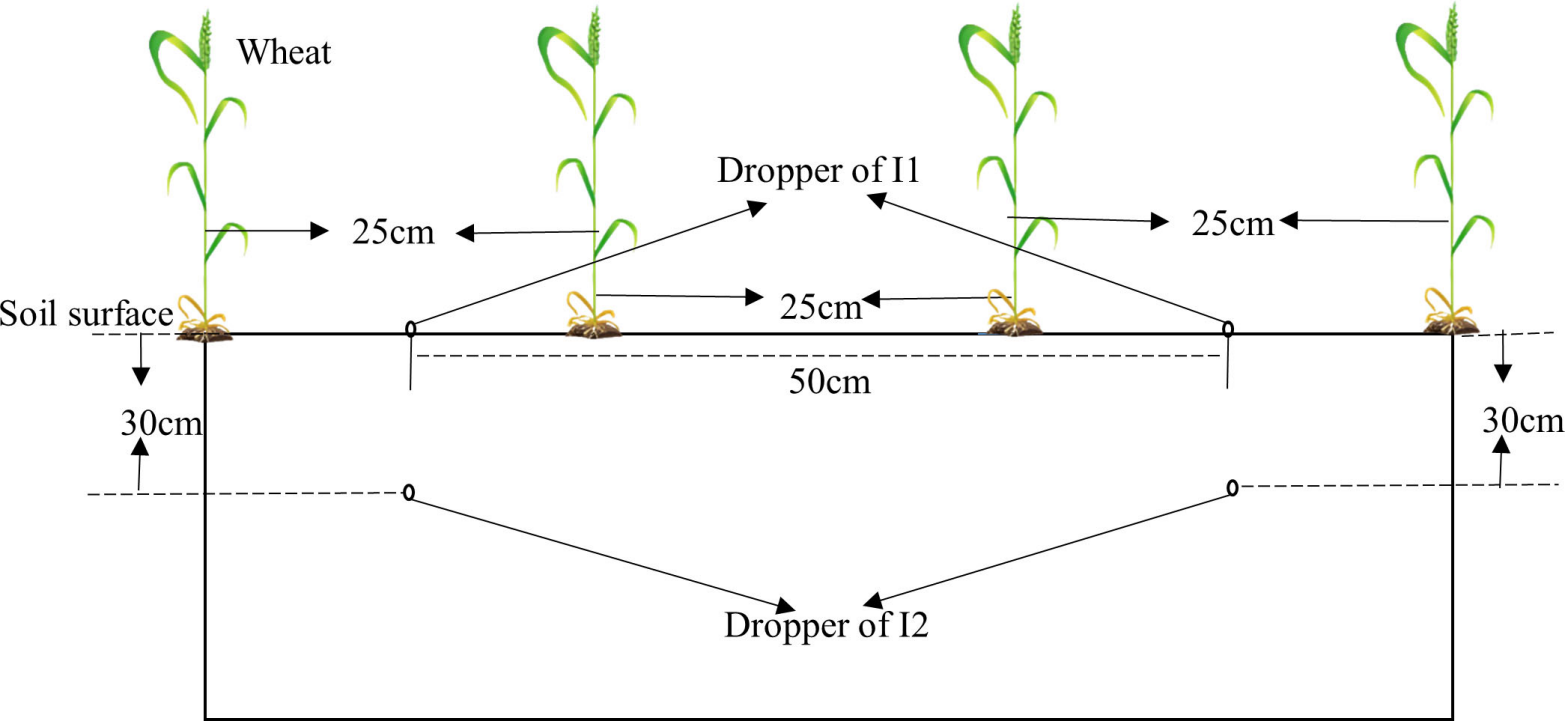
The planting pattern and dropper distribution diagram. CK: traditional irrigation and fertilization methods; I1: surface drip fertilization; I2: subsurface drip fertilization.

A 2-yr field experiment was conducted from October 2020 to June 2022 in the experimental field of Henan Polytechnic University. **Figure 1** is meteorological information for each growing season. The soil of the experimental field is clay loam with an average soil bulk density of 1.32 g·cm^-3^ and field water capacity (FWC) of 27.2% (gravimetrically). Soil nutrient contents from the tillage layer (0–30cm) were total nitrogen (N) 1.02 g kg^-1^, phosphorus 0.86g kg^-1^, and organic matter 28.6 g kg^-1^. The cultivar of winter wheat (*Triticum aestivum* L.) in the experiment was “Pingan11″, which is widely planted in the study area. The experiment involves three irrigation and fertilization modes: traditional irrigation and fertilization method (surface flooding irrigation + artificially applying fertilizer, as CK), surface drip fertilization (I1) and underground drip fertilization (I2). The inner diameter of drip pipe is 15.9 mm, the diameter of drip hole is 0.31mm, and the spacing is 0.5m. Drip pipes for I2 are buried at 0.3m underground (**Figure 2**). Each irrigation mode consisted of 6 replicated plots with an area of 12 m^2^ (5m×2.4m), including 3 nitrogen-applying plots and 3 non-nitrogen-applying plots. In addition, a bare micro-zone was set up to measure soil respiration in each nitrogen-applying plot, in which no wheat was sown. In this micro-zone, a galvanized plate frame with 40cm long, 40cm wide and 120cm high was used to isolate the surrounding wheat roots. All plots were arranged completely randomly in the field. Winter wheat were sown at a sowing rate of 180 kg ha^-1^. The basal fertilizers consisting of N (100 kg ha^-1^), P (60 kg ha^-1^) and K (48 kg ha^-1^) was applied before sowing. 40 kg ha^-1^ of N was applied at jointing and flowering stages respectively by artificially applying fertilizer in the CK and drip fertilization in I1 and I2. Plants were irrigated 60mm ha^−1^ at seedling stage and irrigated 70mm ha^-1^ at jointing and flowering stage respectively.

### 2.2 Experimental indicators and methods

#### 2.2.1 Measurement of plant height, leaf area, tiller number and root shoot biomass

Population characteristics of plants such as plant height, leaf area and tiller number were measured at the jointing and flowering stage respectively. After measuring the population characteristics of plants, the root samples were collected at 20 cm intervals to 80 cm soil depth using a root drill at the jointing and flowering stage respectively. Two root samples were collected in each plot, one centered over the row and the other at the mid-point between the rows.

#### 2.2.2 Measurement of ground evaporation between plants

Ground evaporation between plants were measured 1 to10 days after irrigation at jointing and flowering stage. 3 *in-situ* soil samples with 50mm height were taken from wheat rows using a ring cutter and the bottom of the samples were smoothed to block the bottom flux. After weighing, the samples were placed back in the original position between wheat rows. After that, the soil samples in the ring cutter were weighed every day. After each weighing, 3 soil samples were removed from the ring cutter, and 3 new soil samples were taken from the wheat rows and placed back in the field after weighing.

#### 2.2.3 Photosynthetic rate (Pn) and the instantaneous water use efficiency (IWUE)

At the jointing and flowering stages, Pn and Tr of wheat were measured using a LI-6400XT Portable Photosynthesis System (LI-Cor, Inc., Lincoln, NE, USA) from 9:00am to 11:00am. IWUE of wheat is calculated using the following formula:


(1) $$IWUE=\frac{Pn}{Tr}$$

Where: Pn is the net photosynthetic rate [mmol/(m^2^ s)]; Tr is the transpiration rate [mmol/(m^2^ s)].

#### 2.2.4 Root respiration rate, and root system efficiency

At jointing stage and flowering stage, the root respiration rate was measured immediately after Pn measurement. The plants were first excised along the ground before the root respiration measurements and then the total soil respiration of each treatment was measured with a soil respirometer (SRC-1 with EGM-4, PP-Systems, Boston, USA). In each plot, soil respiration was measured for 3 times on and between rows respectively, and their average value was the total soil respiration of the plot. The bare soil respiration was then measured in three reserved bare micro-zones. The root respiration (R_root_) of each plot was estimated according to the following formula:


(2) $$R_{root}=R_{\text{T}}-R_{\text{S}}$$

Where R_T_ is the total soil respiration of each plot, R_S_ is average value of soil respiration in three reserved bare plots.

Root system efficiency (RE) reflects the relationship between root carbon consumption and photosynthetic carbon sequestration (Ma et al., 2018), which was estimated according to the following formula:


(3) $$\text{RE}=\text{Pn}/{\text{R}}_{\text{root}\cdot }$$

#### 2.2.5 Root activity and chlorophyll fluorescence parameters after flowering

Chlorophyll fluorescence parameters of flag leaf were measured using the Imaging-PAM leaf fluorescence analyser from Walz at 6 days intervals after flowering. After the measurement of chlorophyll fluorescence parameters, roots in the 0-30cm soil layer were collected immediately and put into an ice box and brought back to the laboratory. Root activity was determined using the TTC colourimetric method.

#### 2.2.6 Post-flowering accumulated dry matter (ADM) and pre-flowering dry matter remobilization (DMR)

Plant samples of 0.5 m^2^ in each plot were randomly collected at flowering and maturity stages. The plant samples were dried at 75°C to constant weight and weighed. ADM and DMR were calculated according to the following formulas:


(4) ADM=DMWM–DMWA

(5) DMR=DMWA−(DMWM−grain yield)

Where DMWA is dry matter weight of plants at anthesis, DMWM is dry matter weight of plants at maturity.

#### 2.2.7 Yield traits and water productivity

At maturity, plants of 1.0m^2^ were randomly collected from each plot to measure their yield traits such as spike number, kernels per spike, thousand kernel weight and grain yield. Water productivity (WP), irrigation water productivity (IWP), nitrogen partial factor productivity (NPP), and nitrogen agronomy efficiency (NAE) were also calculated.


(6) $$WP\;(kg\;ha^{-1}mm^{-1})=Y\;(kg\;ha^{-1})/ET\;(mm)$$

(7) $$IWP\;(kg\;ha^{-1}mm^{-1})=Y(kg\;ha^{-1})/IR\;(mm)$$

where ET is the total evapotranspiration, and IR was the recorded irrigation volume throughout the growing season.


(8) $$NPP\;(kg/kg)=Y_{N}/AN$$

(9) $$NAE\;(kg/kg)=(Y_{N}-Y_{NN})/AN$$

Y_N_ is grain yield in N -applying plot; Y_NN_ is grain yield in non-nitrogen-applying plot; AN is nitrogen application amount in N-applying plot.

### 2.3 Statistical analysis

Statistical analyses were performed using the SPSS 25.0 and the differences among different irrigation modes were compared using the least significant difference (LSD) tests (p< 0.05).

## 3 Results

### 3.1 Population characteristics of plants

Different irrigation and fertilization modes had various effects on the population traits of winter wheat (**Table 1**). At the jointing stage, I1 demonstrated significantly higher plant height, leaf area, and tiller number compared to CK, while I2 showed the opposite effect. At the flowering stage, the plant height, leaf area and tiller number of I1 were also higher than that of CK, the plant height and tiller number of I2 were significantly lower, and but the leaf area was higher than that of CK. Furthermore, different irrigation modes caused variations in the root distribution of wheat (**Table 2**). At the jointing stage I1 displayed a significantly larger root system in the top soil layer (0-20cm), while I2 exhibited smaller root system than CK. At the 20-40 cm soil layer, I2 showed a larger root system, but I1 resulted in a smaller system than CK. At 40-80 cm soil layer, the root system of I1 was significantly smaller than that of CK and I2. At the flowering stage, I1 had smaller root system in all soil layers, whereas I2 had smaller in the top layer and but larger in the deeper layer compared to CK. Additionally, at the jointing stage, shoot weight and root weight were higher in I1 and lower in I2 compared to CK (**Table 3**). At the flowering stage, root weight was lower in both I1 and I2, but shoot weight was higher compared to CK, leading to a significant decrease in the root-shoot ratio.

**Table 1:** Population characteristics of winter wheat under different treatments.

Treatments	Jointing stage			Flowering stage		
	Plant height (cm)	Leaf area (cm^2^ per plant)	Number of tillers (per m^2)^	Plant height (cm)	Leaf area (cm^2^ per plant)	Number of tillers (per m^2^)
CK	36.4b	74.6b	1004.3b	75.8a	84.2c	901.4a
I1	38.6a	76.4a	1208.6a	76.1a	87.6b	949.7a
I2	33.8c	70.5c	960.7c	72.5b	92.8a	861.4b

Different letters within a column imply significant differences between different treatments at P< 0.05. CK: traditional irrigation and fertilization methods; I1: surface drip fertilization; I2: subsurface drip fertilization.

**Table 2:** The root distribution characteristics (g m^-2^) of winter wheat under different treatments.

	Jointing stage			Flowering stage		
Treatments	0-20	20-40	40-80	0-20	20-40	40-80
CK	182.2b	51.3b	2.8a	242.8a	79.8a	5.1a
I1	198.3a	49.2c	1.7b	232.6b	74.3b	4.4b
I2	176.8c	53.4a	2.7a	218.3c	78.6a	5.9a

Different letters within a column imply significant differences between different treatments at P< 0.05. CK: traditional irrigation and fertilization methods; I1: surface drip fertilization; I2: subsurface drip fertilization.

**Table 3:** The root and shoot biomass of winter wheat under different treatments.

Treatments	Jointing stage			Flowering stage		
	Root biomass (g m^-2^)	Shoot biomass (g m^-2^)	Root - shoot ratio	Root biomass (g m^-2^)	Shoot biomass (g m^-2^)	Root - shoot ratio
CK	236.3b	842.34b	0.28	327.7a	1505.33b	0.22a
I1	249.2a	899.29a	0.28	311.3b	1642.29a	0.19b
I2	228.9c	780.35c	0.29	302.8c	1698.39a	0.18b

Different letters within a column imply significant differences between different treatments at P< 0.05. CK: traditional irrigation and fertilization methods; I1: surface drip fertilization; I2: underground drip fertilization.

### 3.2 Evapotranspiration and IWUE of plants

**Figure 3:**
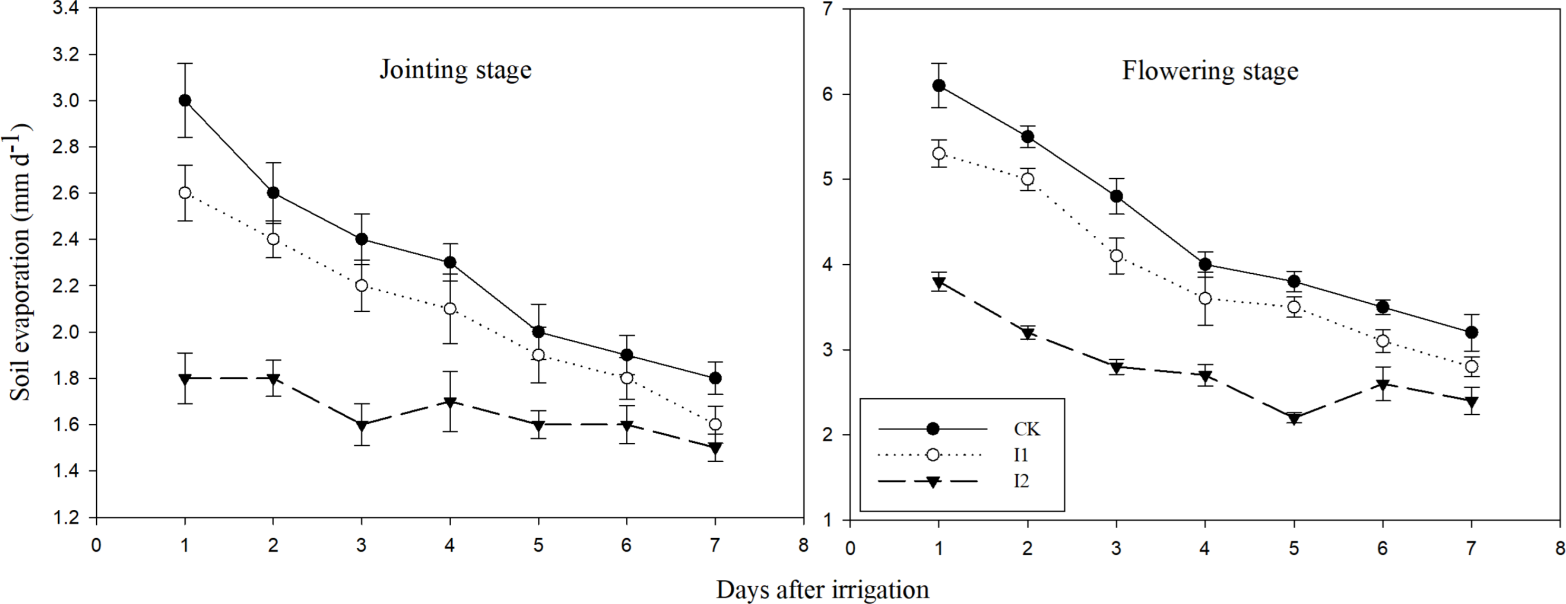
Inter-plant evaporation of winter wheat under different treatments. CK: traditional irrigation and fertilization methods; I1: surface drip fertilization; I2: subsurface drip fertilization. Error bars denote the standard errors of the mean across the treatments (n =3).

Evapotranspiration involves two processes, namely soil evaporation and plant transpiration. **Figure 3** depicts the daily variation of ground evaporation between plants after irrigation at the jointing and flowering stages. Ground evaporation between plants for all treatments decreased gradually with the increase of days after irrigation, but both I1 and I2 showed lower ground evaporation compared to CK. It is clear that two drip irrigation methods were effective in reducing the crop’s ineffective water consumption by lowering ground evaporation between plants. At the jointing stage, Pn and Tr of I1 was similar to CK 7 days after irrigation. Consequently, IWUE of I1 was also the same as CK (**Table 4**). However, I2 had higher IWUE compared to CK, despite significantly lower Pn and Tr. At the flowering stage, I1 had similar Tr and higher Pn compared to CK 7 days after irrigation, leading to a significantly higher IWUE. I2 had similar Pn to I1, but reduced Tr, so I2 had significantly higher IWUE compared to I1. It can be seen that two drip fertilization methods can improve IWUE of crops by regulating Pn and Tr of leaves.

**Table 4:** Pn, Tr and IWUE of winter wheat under different drip irrigation treatments.

Treatments	Jointing stage			Flowering stage		
	Pn (μmolm^-2^ s^-1^)	Tr (μmolm^-2^ s^-1^)	IWUE (Pn/Tr)	Pn (μmolm^-2^ s^-1^)	Tr (μmolm^-2^ s^-1^)	IWUE (Pn/Tr)
CK	13.1b	2.5b	5.2c	24.8b	9.47a	2.63c
I1	14.5a	2.8ab	5.2b	26.1a	9.12a	2.9b
I2	11.8c	2.0c	5.9a	26.7a	8.40b	3.2a

Different letters within a column imply significant differences between different treatments at P< 0.05. CK: traditional irrigation and fertilization methods; I1: surface drip fertilization; I2: underground drip fertilization.; IWUE: instantaneous water use efficiency.

### 3.3 Root activity and chlorophyll fluorescence parameters after flowering

**Figure 4:**
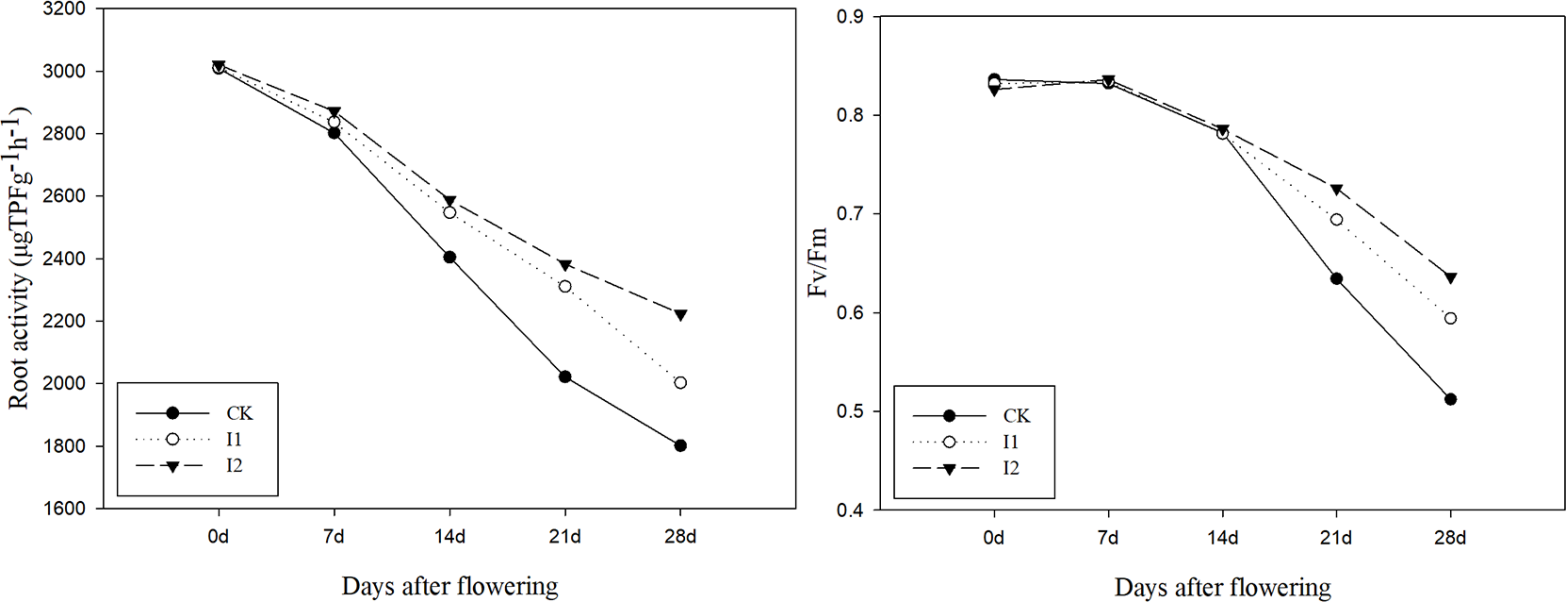
Post flowering Root activity and chlorophyll fluorescence parameters of winter wheat under different treatments. CK: traditional irrigation and fertilization methods; I1: surface drip fertilization; I2: subsurface drip fertilization.

Post-anthesis root activity and photosynthetic capacity can directly affect shoot production and are key indicators reflecting crop senescence. **Figure 4** demonstrates that the root activity of plant in the two drip fertilization modes was higher than that of CK from 14 days after flowering. Fv/Fm is the maximum photochemical quantum yield of photosynthesis PS II reaction center of plant, which can reflect the photosynthetic capacity of leaves. Analysis of chlorophyll fluorescence parameters (Fv/Fm) of winter wheat at 0, 7, 14, 21, and 28 days after flowering revealed that both I1 and I2 increased the Fv/Fm of flag leaves at 21 days after flowering, indicating that drip fertilization improved the photosynthetic performance of plant during the later growth stage of wheat. Post-flowering root activity and Fv/Fm of flag leaf suggested that drip fertilization delayed plant senescence.

### 3.4 Root respiration rate (R_root_), root system efficiency (RE), ADM and DMR

**Figure 5:**
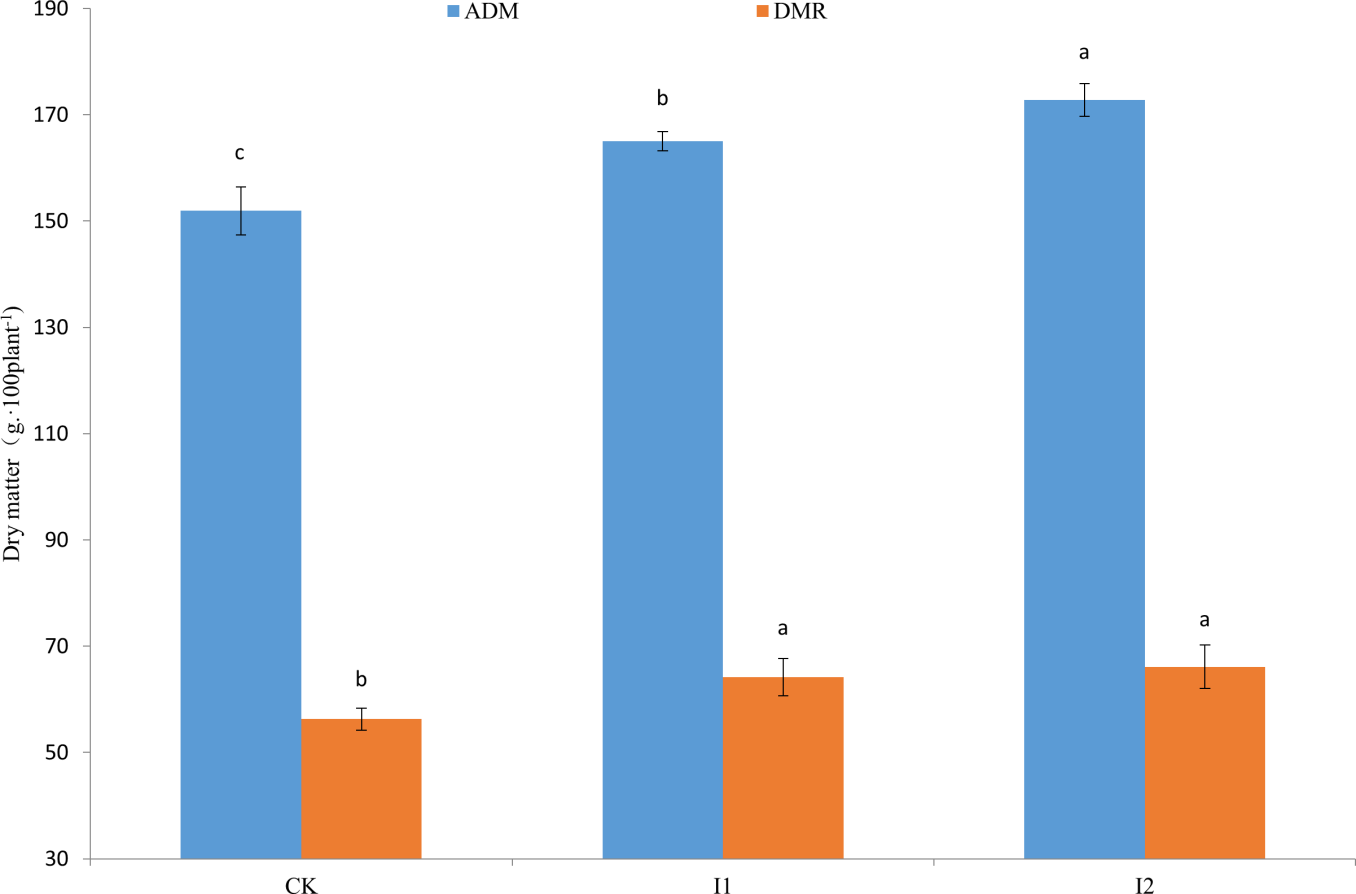
Post-flowering accumulated dry matter (ADM) and pre-flowering dry matter remobilization (DMR) of winter wheat under different treatments.

Different irrigation and fertilization methods had various effects on R_root_ and RE of winter wheat (**Table 5**). At the jointing stages, R_root_ of winter wheat showed CK >I1 > I2. Both I1 and I2 had significantly higher RE compared to CK. But there were no significant differences in RE between I1 and I2. At the flowering stages, both I1 and I2 also reduced R_root_ and enhanced RE compared to CK. I2 had similar Pn, but reduce R_root_ compared to I1, so RE of I2 was significantly higher than that of I1. Different irrigation and fertilization methods also affect ADM and DMR by affecting RE. ADM and DMR of I1 and I2 were significantly higher than those of CK. In particular, the ADM of I2 after flowering was significantly higher than that of I1 and CK (**Figure 5**).

**Table 5:** Effect of different drip irrigation treatments on root respiration rate and root efficiency of winter wheat.

Treatments	Jointing stage			Flowering stage		
	Pn (μmolm^-2^ s^-1^)	R_root_ (μmolm^-2^ s^-1^)	RE (Pn/Rroot)	Pn (μmolm^-2^ s^-1^)	R_root_ (μmolm^-2^ s^-1^)	RE (Pn/R_root_)
CK	13.1b	0.36a	36.4b	24.8b	0.54a	46.1c
I1	14.5b	0.32b	45.3a	26.1a	0.48b	54.4b
I2	11.8a	0.26c	45.4a	26.7a	0.44c	60.7a

Different letters within a column imply significant differences between different treatments at P< 0.05. CK: traditional irrigation and fertilization methods; I1: surface drip fertilization; I2: underground drip fertilization. R_root_: root respiration; RE, root system efficiency.

### 3.5 Yield traits and water and N use efficiency

Different irrigation and fertilization methods had various effects on yield traits of wheat. The number of spikes and grains per spike in I2 and I1t were significantly higher than those of CK (**Table 6**). There was no significant difference in 1000-grain weight among all treatments, but the yield showed the order of I2 > I1 > CK. Furthermore, the drip fertilization optimized the use of water and nitrogen resources, leading to higher IWP, WP, NPP and NAE than those of CK (**Table 7**). These findings suggested that two drip fertilization methods had significant effects on the yield traits and absorption and utilization of water and nitrogen, with I2 resulting in the highest grain yield, WP, WP, NPP and NAE.

**Table 6:** Yield characteristics of wheat under different treatments.

Years	Treatments	Spikes number (10^4^ha^-1^)	Grains per spike	1000 Kernel weight (g)	Y_N_ (kg.ha^-1^)	Y_NN_ (kg.ha^-1^)
2020-2021	CK	653.4b	32.2b	36.4a	7916.9c	6712.7c
	I1	665.4a	34.7a	36.5a	8115.2b	6813.8b
	I2	676.6a	33.6ab	36.6a	8497.8a	7113.6a
2021-2022	CK	658.4b	32.0b	35.7a	8078.7c	6942.4c
	I1	679.6a	34.9a	36.1a	8317.3b	7103.2b
	I2	697.2a	34.6a	36.7a	8500.9a	7211.5a

Different letters within a column imply significant differences between different treatments at P< 0.05. CK: traditional irrigation and fertilization methods; I1: surface drip fertilization; I2: underground drip fertilization; Y_N_: grain yield in N -applying plot; Y_NN_: grain yield in non-nitrogen-applying plot.

**Table 7:** Water and nitrogen utilization efficiency of different treatments.

Years	Treatment	Irrigation water (mm)	Nitrogen application(kg.ha^-1^)	ET (mm)	IWP (kg ha^-1^ mm^−1^)	WP (kg ha^-1^ mm^−1^)	NPP (kg.kg^−1^)	NAE (kg.kg^−1^)
2020-2021	CK	200	180	382.2a	39.6c	20.7c	43.9c	6.7c
	I1	200	180	342.7b	40.6b	23.7b	45.1b	7.2b
	I2	200	180	339.6c	42.5a	25.0a	47.2a	7.7a
2021-2022	CK	200	180	387.9a	40.4c	20.8c	44.9c	6.3c
	I1	200	180	339.8b	41.6b	24.5a	46.2b	6.7b
	I2	200	180	340.9b	42.5a	24.9a	47.2a	7.2a

Different letters within a column imply significant differences between different treatments at P< 0.05. CK: traditional irrigation and fertilization methods; I1: surface drip fertilization; I2: underground drip fertilization. WP, water productivity; IWP, irrigation water productivity; NPP, nitrogen partial productivity; NAE, nitrogen agronomic efficiency.

## 4 Discussion

### 4.1 Effects of different drip fertilization methods on evapotranspiration and IWUE in winter wheat

Water use efficiency of crops is mainly affected by evapotranspiration (Xu et al., 2018). Evapotranspiration encompasses evaporation from the soil surface and transpiration from plants (Li et al., 2010; Wu and Bao, 2015). Soil surface evaporation, an inefficient mode of water consumption, constitutes a significant portion of crop’s water consumption. In the case of winter wheat, approximately 30% of water consumption during the entire growing period is attributed to ground evaporation between plants (Liu et al., 2002). Consequently, reducing ground evaporation between plants is crucial for conserving irrigation water and enhancing water use efficiency of crop. Ground evaporation between plants is inextricably linked to Surface soil water content, with lower surface soil water content leading to increased resistance to soil evaporation (Gao et al., 2008). Irrigation methods directly impact ground evaporation between plants by altering surface soil water content. Two drip irrigation treatments examined in this study significantly diminished ground evaporation and curtailed inefficient water consumption, ultimately benefiting water use efficiency of crop. I2, which directly provides water to the deeper roots of plants while maintaining a relatively dry surface soil, mitigates ineffective evaporation from the soil surface and ensures that the majority of water is utilized by plant roots (Elmaloglou and Diamantopoulos, 2009). Thus, I2 leads to substantially lower ground evaporation and markedly higher IWP compared to I1.

Different drip irrigation methods can also improve the IWUE of crop by regulating the Pn and Tr of leaves. At the flowering stage, I1 had similar Tr and higher Pn compared to CK, and I2 significantly reduced Tr and increased Pn, so their IWUE were also markedly higher compared to CK. The physiological water-saving mechanisms of different irrigation methods came from the relationship between Pn, Tr and stomatal opening of plant. The relationship between plant Tr and stomatal conductance was linear, while there is a gradual saturation relationship between Pn and stomatal conductance. By moderately reducing stomatal conductance, transpiration water consumption can be significantly decreased without notably impacting Pn (Tang et al., 2005). Thus, Pn and Tr can be regulated by controlling soil moisture to influence stomatal opening of plant. For instance, when crops undergo alternate furrow irrigation, roots in the humid zone absorb water to supply the normal growth of crops, while roots in the dry zone generate drought signals to regulate stomatal opening and reduce luxury transpiration without reducing the accumulation of photosynthate, thereby achieving the purpose of physiological water saving (Shahnazari et al., 2007; Du et al., 2008; Shu et al., 2020). The present study demonstrated that Pn of I2 was similar to that of I1, but its Tr was notably lower, resulting in an a higher IWUE compared to I1. This is primarily attributable to the difference in the vertical spatial distribution of soil water between I2 and T1. The drought signal from roots in the surface dry zone was transmitted to leaves to regulate stomatal opening under I2, while roots in the lower wet zone absorbed water from the soil to meet the needs of crops, so as to optimize the relationship between Pn and Tr and improve IWUE of leaf.

### 4.2 Impact of drip fertilization on root activity and chlorophyll fluorescence parameters

In addition to genetic factors, growth, development, and distribution of the root system are primarily influenced by soil environmental conditions such as water and fertilizer (Zhuge et al., 2004; Ma et al., 2022). Soil water and fertilizer distribution considerably affects the microenvironment of the soil profile (Wang et al., 2009), which subsequently influences the physiological characteristics of both the root and shoot. Compared with surface flood irrigation, drip fertilization can maintain higher level of soil water and fertilizer around the drip head for a long time, and make the water and fertilizer continuously supplement to the root zone, providing more favorable environmental conditions for crops, thus affecting the physiological characteristics of roots (Raviv et al., 1999). After flowering, the root activity of wheat began to decline, but in this study, the decrease range of root activity in two drip fertilization treatments was significantly less than that of CK, which affected the physiological characteristics of shoot by influencing the absorption of water and nutrients by roots. Consequently, Fv/Fm of the flag leaf of wheat was significantly higher in I1and I2 than that in CK. Fv/Fm reflects the biological activity of the PS II reaction center in plant leaves. Post-flowering root activity and photosynthetic capacity directly impact crop production and serve as vital indicators of crop senescence. Yu et al. (2015) demonstrated that compared to surface drip irrigation, subsurface drip irrigation could maintain high root activity and protective enzyme activity in deep soil, and improve the photosynthesis and fluorescence characteristics of plants in late growth period. The post-flowering root activity and Fv/Fm in this study also suggested that two drip fertilization delayed crop senescence, especially I2 had higher root activity and Fv/Fm than I1, which is crucial for promoting grain filling after anthesis.

### 4.3 Impact of drip fertilization on root and shoot characteristics and post-flowering ADM

Different irrigation and fertilization methods influence the physiological and ecological characteristics of root and shoot by regulating the distribution of water and nutrient, ultimately affecting yield formation (Singh et al., 2016). During the early growth stages of crop, subsurface drip irrigation restricted the upward movement of soil water and somewhat inhibited shoot growth, and promoted the root system to go deep down. In the later growth stage, subsurface drip irrigation increased the underlying root system and enhanced root activity, promoted the absorption of water and nutrients in the deep layer, and thus improved the morphological characteristics and physiological activities (e.g., plant height and leaf area, photosynthetic rate) of plants (Araki and Iijima, 2005), and promote dry matter accumulation in later growth stages compared to surface drip irrigation (He et al., 2003). In this study, during the early stages of crop, roots were primarily distributed in the shallow soil, and I2 had lower surface soil moisture than I1 and CK, limiting plant growth. Consequently, I2 had significantly lower plant height, leaf area, and shoot biomass compared to CK and I1. With the extension of roots in the later stages of growth, I2 had more roots in the deep soil, which could absorb more soil water and nutrients to supply plant growth and promote dry matter accumulation. Therefore, shoot biomass of I2 was significantly higher than that of CK and I1 at maturity stage. In addition, in order to maintain the physiological and metabolic functions of the root system, the root system also consumes a lot of photosynthates through root respiration (McCree, 1986). Therefore, on the basis of maintaining the stability of leaf photosynthesis, reducing the carbon consumption by root respiration is conducive to improving crop yield (Liu and Li, 2005). Soil water had significant effects on leaf photosynthesis (Ma et al., 2015) and root respiration (Paudel et al., 2018). Appropriate soil moisture can improve crop yields by optimizing the allocation of photosynthates between the root and shoot (Mingo et al., 2004). Therefore, it is reasonable to reduce root respiration and optimize the allocation of photosynthates between the root and shoot by controlling soil moisture, ultimately improving wheat yield. This study showed that I1 and I2 increased leaf Pn, decreased root respiration and root weight, optimized the allocation of photosynthates between the root and shoot, and thus increased dry matter accumulation of shoot. Notably, the ADM of I2 after flowering was significantly higher than that of I1. Additionally, grain growth during grain-filling depends on both current photosynthesis and re-mobilization of pre-flowering photosynthates into the grain (Foulkes et al., 2002; Richards et al., 2002), and the remobilization of pre-flowering photosynthates is also crucial for wheat yield especially under water stress (Richards et al., 2002). This study also revealed that the pre-flowering DMR was significantly higher in both I1 and I2compared to CK.

### 4.4 Effect of drip fertilization on yield traits and utilization of water and nitrogen

Different irrigation and fertilizer methods (by affecting water and fertilizer distribution in soil profiles) not only reduce water consumption by plants, but also affect grain yield by affecting the photosynthetic capacity of crops (Jha et al., 2017; Zhang et al., 2017; Jha et al., 2019). Compared with surface drip irrigation, underground drip irrigation not only reduces ineffective water consumption by keeping the surface dry and reducing ineffective evaporation, but also maintains a higher soil water content in the root zone, thus leading to an increase in crop yield (Badr et al., 2010). The formation and improvement of crop yields largely depend on the ability of crops to utilize deep soil water and nutrients (Miao et al., 2002). Optimization of root distribution and enhancement of root absorption capacity are key to improving the utilization of water and nutrients in deep soil (Wang et al., 2001). Compared with surface irrigation, subsurface drip irrigation promotes the migration of water and nutrients to the deeper soil (Wang et al., 2011), which induces the root system to extend to the lower layer and fully absorb the water and nutrients in the deeper soil, contributing to the improvement of crop yield and the utilization efficiency of water and fertilizer (Zhang et al., 2011; Cutforth et al., 2013). This study also proved that I2 increased the uptake and utilization of water and fertilizer by improving root vitality and optimizing root distribution, thus improving post-flowering morphological and physiological characteristics of plants (higher Pn and leaf area) compared with CK and I1. Higher Pn and effective photosynthetic leaf area are the prerequisite for obtaining higher photosynthates. Therefore, compared with CK and I1, I2 improved grain yield and WP of crop.

In addition, drip fertilization can apply water and fertilizer to crop root quantitatively and accurately, significantly reducing nitrogen leaching loss, and thus improving nitrogen use efficiency (Huang et al., 2008; Jayakumar et al., 2015). Different drip fertilization methods also had various effects on nitrogen uptake and utilization. Compared with surface drip irrigation, underground drip irrigation can increase the absorption and utilization of nitrogen by crops, thus reducing the leaching of nitrate nitrogen (Kong et al., 2010) and improving the utilization efficiency of water and fertilizer (Wang et al., 2009). This study also showed that two drip fertilization modes significantly increased the grain yield and the utilization efficiency of water and nitrogen compared to CK, especially I2 had the highest grain yield and water/nitrogen use efficiency.

## 5 Conclusion

Drip fertilization significantly affected the population and physiological characteristics of winter wheat. I1 increased plant height, leaf area and tiller number throughout the growing period, while I2 decreased plant height and tiller number. I2 decreased the leaf area in the early stage, but increased it in the late stage. The different irrigation modes also affected the root distribution of wheat. At the flowering stage, I1 resulted in a lower root system in all soil layers, I2 led to a lower upper root system, but a higher deeper root system compared to CK. Both I1 and I2 also reduced total root weight and root-shoot ratio of wheat. I1 improved IWUE by increasing Pn of wheat. I2 improved IWUE by reducing Tr of wheat. Both I1 and I2 increased the root activity, photosynthetic capacity and RE of wheat at the later growth stage, which promoted the accumulation of dry matter after anthesis and the increase of grain yield. In addition, since I1 and I2 optimized the utilization of water and nitrogen resources in wheat, their WP, IWP, NPP and NAE were significantly higher than those of CK, notably, I2 had the highest WP, IWP, NPP and NAE.

## Data availability statement

The raw data supporting the conclusions of this article will be made available by the authors, without undue reservation.

## Author contributions

StM and ScM contributed to conception and design of the study. YM organized the database and performed the statistical analysis. StM wrote the first draft of the manuscript. YM and QH edited the manuscript. All authors contributed to the article and approved the submitted version.
